# Crystal structure of poly[hexa-μ-bro­mido-bis{2-[1-(py­ri­din-2-yl)ethyl­idene­amino]ethanol­ato}tetracopper(II)]

**DOI:** 10.1107/S2056989023011040

**Published:** 2024-01-12

**Authors:** Bocar Traoré, Ngoné Diouf, Momath Kébé, Rokhaya Guéye-Sylla, Ibrahima Elhadji Thiam, Ousmane Diouf, Pascal Retailleau, Mohamed Gaye

**Affiliations:** a Département de Chimie, Faculté des Sciences et Techniques, Université Cheik Anta Diop, Dakar, Senegal; b Substances Naturelles, CNRS UPR 2301, Université Paris-Sud, Université, Paris-Saclay, 1 av. de la Terrasse, 91198 Gif-sur-Yvette, France; Universidade de Sâo Paulo, Brazil

**Keywords:** crystal structure, acetyl­pyridine, 2-amino­ethanol, 2-(1-((2-hyroxyeth­yl)imino)­acetyl­pyridine), square pyramidal, tetra­hedral

## Abstract

The reaction of the Schiff base 2-[1-(pyridin-2-yl)ethyl­idene­amino]­ethanol (H*L*), which is formed by reaction of 2-amino­ethanol and 2-acetyl­pyridine with CuBr_2_ in ethanol results in the isolation of the new polymeric complex poly[hexa-μ-bromido-bis­{2-[1-(pyridin-2-yl)ethyl­idene­amino]­ethano­lato}tetra­copper(II)], [Cu_4_Br_6_(C_9_H_11_N_2_O)_2_]_*n*_ or [Cu_4_Br_6_ *L*_2_]_*n*_. The asymmetric unit of the crystal structure of the polymeric [Cu_4_Br_6_ *L*_2_]_*n*_ complex is composed by four copper (II) cations, two monodeprotonated mol­ecules of the ligand, and six bromide anions, which act as bridges. The ligand mol­ecules act in a tridentate fashion through their azomethine nitro­gen atoms, their pyridine nitro­gen atoms, and their alcoholate O atoms. The crystal structure shows two types of geometries in the coordination polyhedrons around Cu^2+^ ions. Two copper cations are situated in a square-based pyramidal environment, while the two other copper cations adopt a tetra­hedral geometry. Bromides anions acting as bridges between two metal ions connect the units, resulting in a tetra­nuclear polymer compound.

## Abstract 2

In the title Schiff base tetra­nuclear copper(II) complex, two discrete environments are present in the structure: CuN_2_OBr_2_ and CuBr_4_. Two copper(II) cations are situated in distorted square-based pyramidal environment, while two copper(II) cations are located in distorted tetra­hedral geometry.

## 1. Chemical context

Schiff bases attract a great attention as ligands due to their simplicity of formation from amino and carbonyl derivatives. A rich coordination variability can be thus easily be attained and profited by following the introduction of other functional groups. Schiff base ligands are becoming increasingly important as they have inter­esting biological activities such as anti­bacterial, anti­tumor, insulin-mimetic and anti­fungi (Patil *et al.*, 2012 ▸; Thompson & Orvig, 2001 ▸), and catalytical properties (Sutradhar *et al.*, 2013 ▸). They are used in the preparation of photo- and pH-responsive sensors (Li *et al.*, 2013 ▸), fluorescent receptors of metals (Chen *et al.*, 2013 ▸), non-linear materials (Massue *et al.*, 2013 ▸), nano-particles (Deng *et al.*, 2013 ▸), hybrid inorganic–organic materials (Bhaumik *et al.*, 2013 ▸), and even uranium complexes (Asadi *et al.*, 2013 ▸), and ionic liquids (Ouadi *et al.*, 2006 ▸). Many related tridentate Schiff base ligands have been successfully employed to build clusters of copper(II) ions bridged by halogen atoms (Wang *et al.*, 2013 ▸; Sall *et al.*, 2019 ▸; Sun *et al.*, 2005 ▸). The incorporation of an amino alcohol fragment generally leads to the formation of [Cu_4_O_4_] cubane-type clusters (Yan *et al.*, 2009 ▸; Xie *et al.*, 2002 ▸). In our present work, we have synthesized and characterized through X-ray diffraction analysis the title tetra­nuclear complex formulated as [Cu_4_Br_6_ *L*_2_]_*n*_, (H*L* = 2-{1-[(2-hydroxy­eth­yl)imino]­acetyl­pyridine}). 
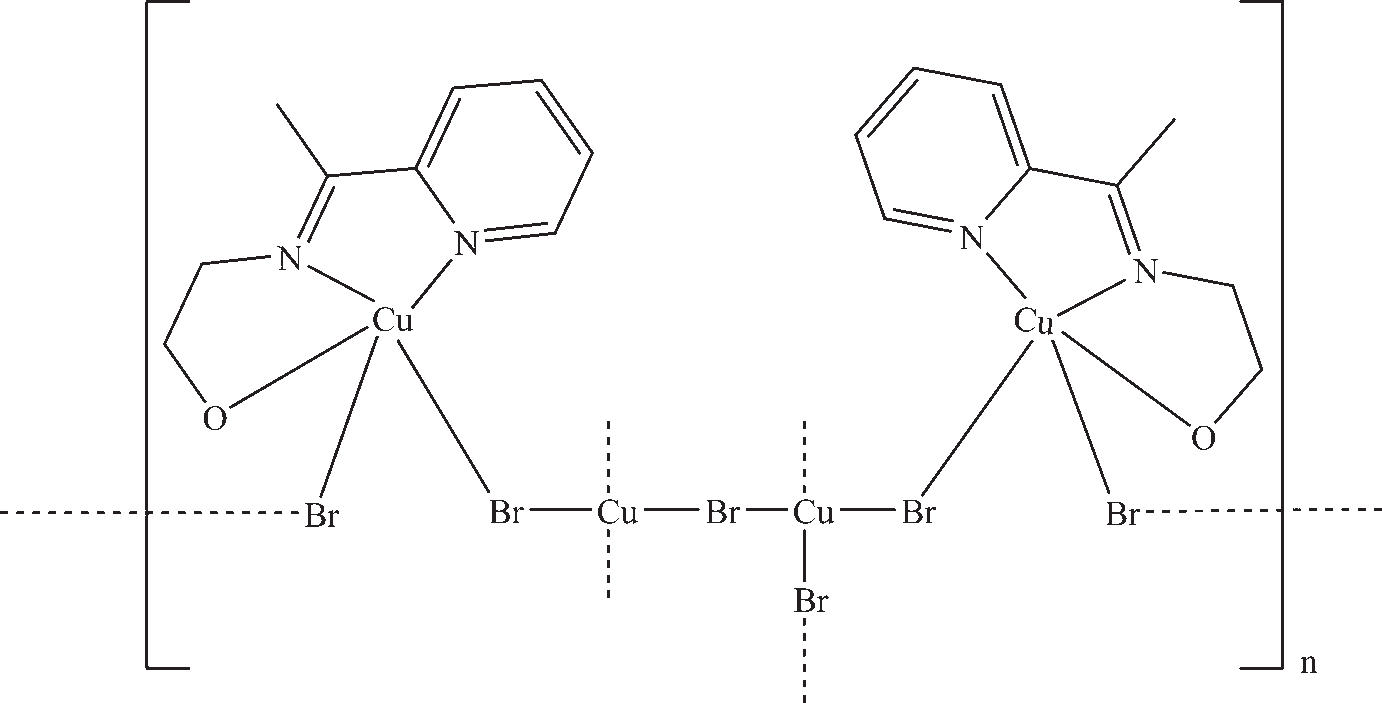


## 2. Structural commentary

**Figure 1:**
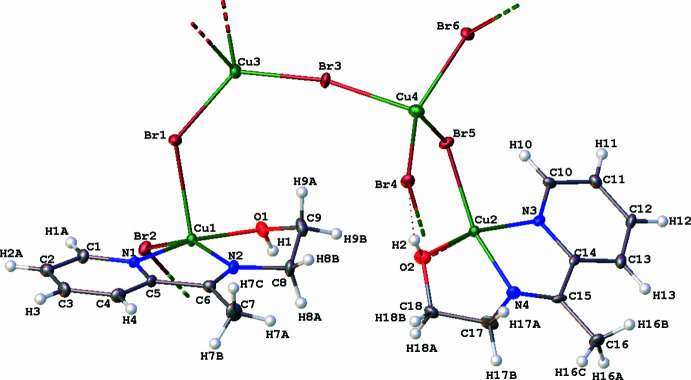
A view of the title compound, showing the atom-labeling scheme. Displacement ellipsoids are plotted at the 30% probability level.

The reaction of acetyl­pyridine and 2-amino­ethanol in 1:1 ratio in ethanol yields the ligand 2-{1-[(2-hydroxyeth­yl)imino]­acetyl­pyridine} (H*L*). The reaction of the ligand H*L* with copper bromide yields a complex in which the ligand is reacted in its deprotonated form as *L* ^−^. The coordination complex is formulated as [Cu_4_ *L*_2_Br_6_]_*n*_ (I) (Fig. 1 ▸).

In the crystal of the tetra­nuclear complex, each of the two deprotonated ligands acts in a tridentate fashion, linking exclusively one copper(II) cation through its imino nitro­gen atom, its pyridine nitro­gen atom and its alcoholate oxygen atom. The two other Cu cations are only coordinated to bromide anions, which act as bridges. The metal centers present two different environments. According to the Addison index (Addison *et al.*, 1984 ▸) *τ* = (*β* − *α*)/60 (*β* and *α* are the largest values of the bond angles around the central atom) the coordination geometry around a penta­coordinated metal center can be discussed: *τ* = 0 describes a perfect square-pyramidal while *τ* = 1 describes a perfect trigonal–bipyramidal geometry. The geometries around the penta­coordinated Cu1 and Cu2 atoms are best described as distorted square-pyramidal, as shown by the Addison index: *τ* = 0.0967 (Cu1) and *τ* = 0.1517 (Cu2). For Cu1, the basal plane is occupied by O1, N1, N2 and Br1, the apical position being occupied by the Br2 atom. For Cu2, the basal plane is occupied by O2, N3, N4 and Br6, the apical position being occupied by the Br5 atom. Additionally, the sums of the angles subtended by the atoms in the basal plane, which are equal to 356.1° (Cu1) and 356.3° (Cu2), deviate severely from the ideal value of 360°. For Cu1 and Cu2, the bond-angle values [92.51 (13)–108.25 (16)°] between the atom occupying the apical position and the atoms in the basal plane also deviate considerably from the ideal value of 90°. Additionally, the *cissoid* bond-angle values [80.5 (2)–98.12 (13)°] also deviate from the ideal value of 90°. The coordination of the ligand to Cu1 or Cu2 results in the formation of two five-membered CuNCCN rings with bite-angle values of 80.5 (2)° (Cu1) and 80.6 (2)° (Cu2) and CuNCCO rings with bite-angle values of 81.9 (2)° (Cu1) and 81.6 (2)° (Cu2). The geometry around the tetra­coordinated atoms Cu3 and Cu4 was determined using the distortion index or the tetra­gonality parameter (Yang *et al.*, 2007 ▸), which is stated as follows: *χ* = (360 − *α* − *β*) / 141 (*α* and *β* are the two largest angles around the central atom). *χ* = 0 designates a perfect square-planar geometry and χ = 1 gives a perfect tetra­hedron. The values of *χ* = 0.88 (Cu3) and *χ* = 0.86 (Cu4) are indicative of distorted tetra­hedral geometries around the metal centers. In fact, the Br—Cu—Br bond-angle values [94.15 (4)–126.29 (6)°] deviate severely from the ideal value of 109.5° for a perfect tetra­hedral geometry.

The Cu—Br_basal plane_ bond lengths (Table 1 ▸) [Cu1—Br1 = 2.3739 (10) Å, Cu2—Br6 = 2.3878 (11) Å] are shorter than the Cu—Br_apical_ bond lengths [Cu1^i^—Br2 = 2.6540 (11) Å, Cu2—Br5 = 2.6357 (11) Å]. These values are in accordance with the Cu—Br bond distances reported in the literature (Jiang *et al.*, 2008 ▸; Godlewska *et al.*, 2011 ▸). An asymmetric bridge behavior of the bromide anion is observed, as shown by the following bond lengths: Cu3—Br3 = 2.3987 (11) Å/Br3—Cu4^ii^ = 2.6288 (12) Å and Cu3—Br3 = 2.3987 (11) Å/Cu3—Br4 = 2.6469 (12) Å. The Cu—N_Py_ bonds [1.992 (5) Å (Cu1—N1) and 1.993 (5) Å (Cu2—N3)] are slightly longer than the Cu—N_imine_ distances [1.964 (5) Å (Cu1—N2) and 1.968 (5) Å (Cu2—N4)]. The Cu—O bond lengths represent the longest distances [2.008 (4) Å (Cu1—O1) and 2.011 (4) Å (Cu2—O2)]. The Cu—N and Cu—O distances are comparable to the values reported for similar complexes (Xue *et al.*, 2010 ▸; Kébé *et al.*, 2021 ▸).

## 3. Supra­molecular features

**Figure 2:**
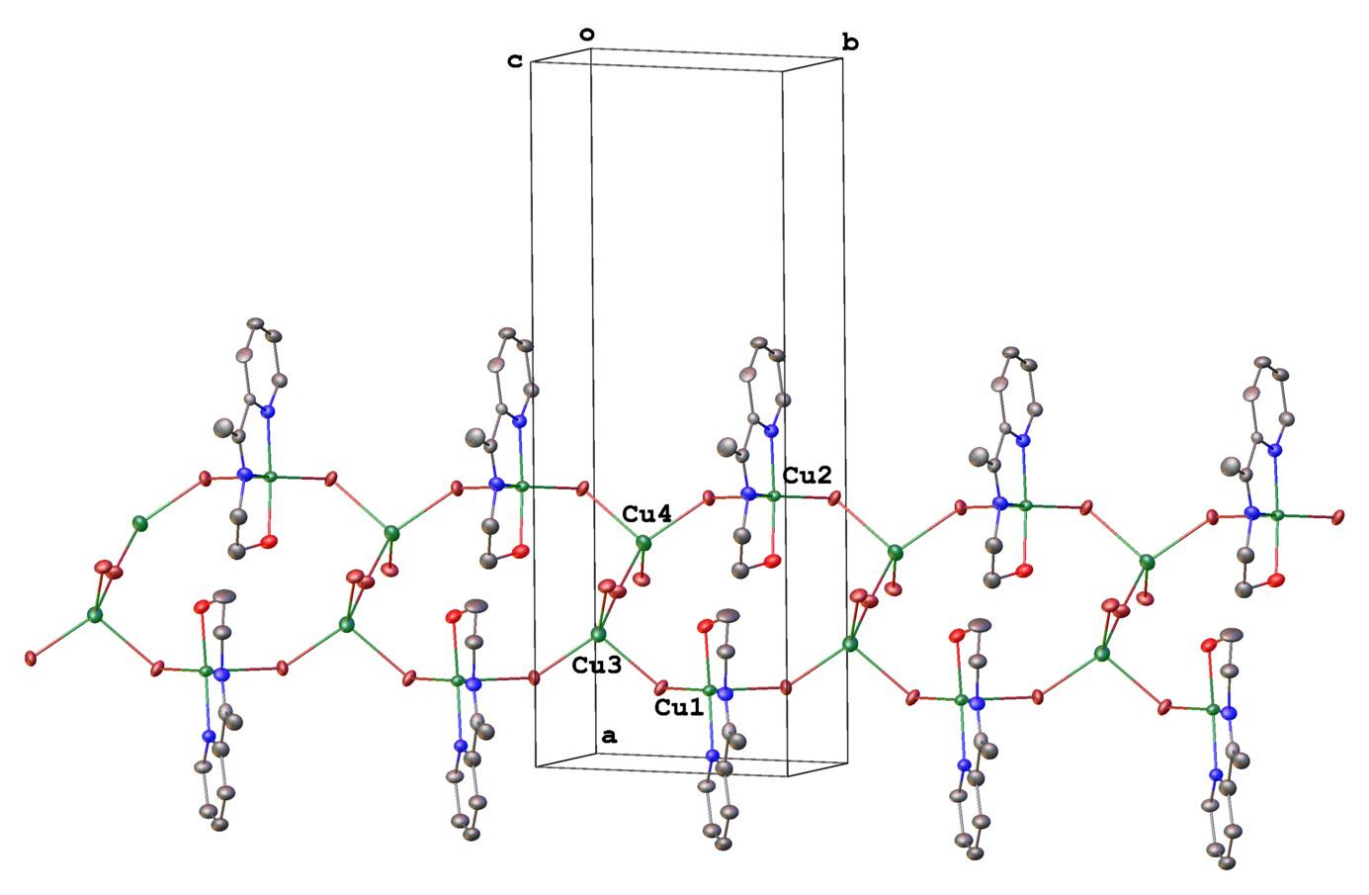
Fragment of a [010] polymeric chain in the crystal structure of the title compound.

**Figure 3:**
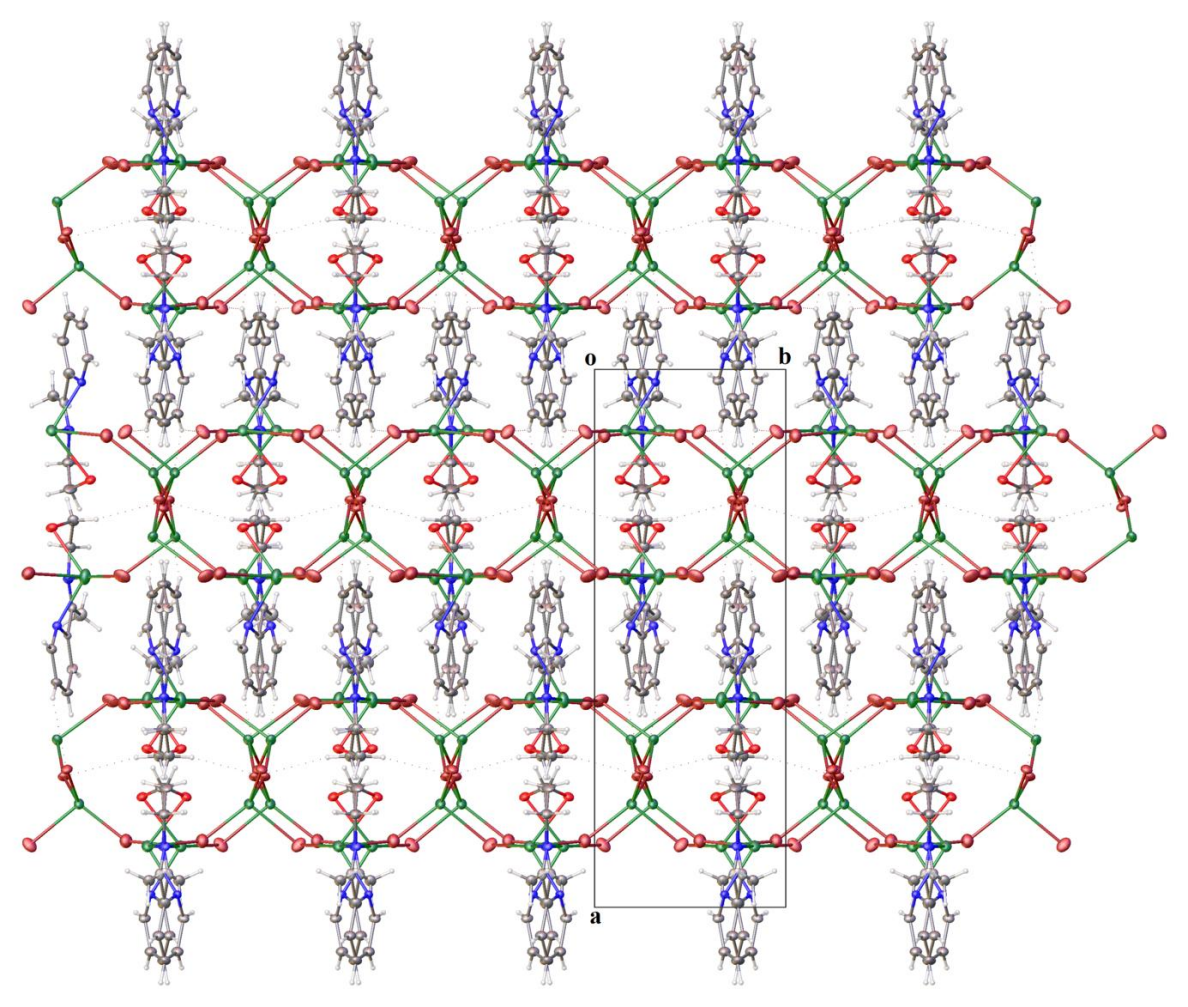
The packing in the crystal of the title complex, viewed along the *c* axis.

The crystal structure shows a three-dimensional polymer complex. The formation of this polymer was facilitated by bromide ions bridging copper(II) ions. The crystal packing of the complex is presented in Fig. 2 ▸. The polymer then develops as a band parallel to the *bc* plane (Fig. 3 ▸). Numerous inter­molecular hydrogen bonds of the type C—H⋯Br (Table 2 ▸) connect adjacent units, resulting in a three-dimensional network.

## 4. Database survey

A search of the CSD (Version 5.42, November 2021 update; Groom *et al.*, 2016 ▸) gave seven hits. One is a mononuclear Mo^5+^ complex (BOFTOH; Jurowska *et al.*, 2014 ▸) and two are coordination dinuclear complexes of Mn^2+^ (JIKLIY and JIKLOE; Brooker & McKee, 1990 ▸). Similar Schiff ligands in which the methyl group is replaced by a phenyl group yielded three mononuclear Ni^2+^ complexes (FOVBIE, FOVBOK, FOVBUQ; Chatterjee *et al.*, 2019 ▸). Another similar ligand in which the alcohol group is replaced by a meth­oxy group yielded a Pd^2+^ complex (PUYQUX; Nyamato *et al.*, 2015 ▸).

## 5. Synthesis and crystallization

To a solution of acetyl­pyridine (0.121 g, 1 mmol) in 10 mL of ethanol, 2-amino­ethanol (0.0610 g, 1 mmol) previously dissolved in 5 mL of ethanol was added. The resulting red solution was refluxed for 2 h. After cooling to room temperature, a solution of CuBr_2_ (1 mmol, 0.2234 g) in 5 mL of ethanol was added. The resulting mixture was stirred for 2 h, and the filtrate was left for slow evaporation. Green crystals suitable for X-ray diffraction were collected after a week. The compound was formulated as [Cu_4_Br_6_ *L*_2_]_*n*_, where (H*L*) is 2-{1-[(2-hyrdoxyeth­yl)imino]­acetyl­pyridine}; Analysis calculated for C_18_H_22_Br_6_Cu_4_N_4_O_2_: C, 20.37; H, 2.06; N, 5.25. Found: C, 20.40; H, 2.09; N, 5.29%. IR (ν, cm^−1^): 3075, 1650, 1622, 1597, 1540, 1430, 1265, 1190, 899, 793. UV–Visible [DMSO, λ_max_ (nm)]: 288, 457, 680. Λ (S cm^2^ mol^−1^): 15.

## 6. Refinement

Crystal data, data collection and structure refinement details are summarized in Table 3 ▸. H atoms were placed in idealized positions and refined using a riding model. The structure was refined considering a positional disorder for the following atoms: Cu1*A*, Br1*A* ,Cu3*A*, Br2*A*, Br6*A*, Cu2*A*, Br5*A*, Cu4*A*, with occupancy of *ca* 0.06–0.08.

## Supplementary Material

Crystal structure: contains datablock(s) I. DOI: 10.1107/S2056989023011040/ex2076sup1.cif

CCDC reference: 2321970

Additional supporting information:  crystallographic information; 3D view; checkCIF report

## Figures and Tables

**Table 1:** Selected geometric parameters (Å, °)

Br2—Cu1^i^	2.6540 (11)	Cu1—N2	1.964 (5)
Br2—Cu3	2.4046 (12)	Cu1—N1	1.992 (5)
Br1—Cu1	2.3739 (10)	Br6—Cu2	2.3878 (11)
Br1—Cu3	2.5098 (11)	Br6—Cu4^iii^	2.5359 (14)
Br4—Cu3	2.6469 (12)	Br5—Cu2	2.6357 (11)
Br4—Cu4	2.3990 (12)	Br5—Cu4	2.4092 (14)
Br3—Cu3	2.3987 (11)	Cu2—N4	1.968 (5)
Br3—Cu4^ii^	2.6288 (12)	Cu2—O2	2.011 (4)
Cu1—O1	2.008 (4)	Cu2—N3	1.993 (5)
			
Br1—Cu1—Br2^iii^	99.38 (3)	N2—Cu1—Br1	155.74 (16)
O1—Cu1—Br2^iii^	97.34 (14)	N2—Cu1—O1	81.9 (2)
O1—Cu1—Br1	95.65 (14)	N1—Cu1—O1	161.5 (2)
N2—Cu1—Br2^iii^	104.87 (16)	N4—Cu2—Br6	152.70 (16)

Symmetry codes: (i) 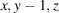 ; (ii) 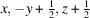 ; (iii) 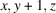 .

**Table 2:** Hydrogen-bond geometry (Å, °)

*D*—H⋯*A*	*D*—H	H⋯*A*	*D*⋯*A*	*D*—H⋯*A*
O1—H1⋯Br3	0.82	2.43	3.240 (4)	172
O2—H2⋯Br4^iii^	0.82	2.40	3.206 (4)	167
C4—H4⋯Br2^iv^	0.93	3.04	3.915 (8)	158
C13—H13⋯Br5^v^	0.93	2.99	3.843 (6)	154
C1—H1*A*⋯Br1	0.93	2.96	3.477 (6)	117
C11—H11⋯Br3^v^	0.93	2.95	3.670 (6)	136
C11—H11⋯Br5^vi^	0.93	3.05	3.790 (7)	138
C9—H9⋯Br5^vii^	0.93	2.98	3.888 (8)	166
C2—H2*A*⋯Br2^viii^	0.93	3.09	3.818 (7)	136
C2—H2*A*⋯Br4^viii^	0.93	2.91	3.658 (7)	139
C18—H18*A*⋯Br2^iii^	0.97	3.02	3.965 (7)	164
C18—H18*B*⋯Br4	0.97	3.13	4.086 (7)	169
C7—H7*C*⋯Br1^vii^	0.96	2.99	3.621 (7)	125
C10—H10⋯Br6	0.93	2.98	3.482 (6)	115
C16—H16*A*⋯Br6^vii^	0.96	2.92	3.636 (7)	133

Symmetry codes: (iii) 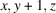 ; (iv) 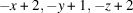 ; (v) 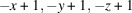 ; (vi) 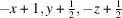 ; (vii) 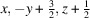 ; (viii) 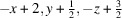 .

**Table 3:** Experimental details

Crystal data	
Chemical formula	[Cu_4_Br_6_(C_9_H_11_N_2_O)_2_]
*M*_r_	1060
Crystal system, space group	Monoclinic, *P*2_1_/*c*
Temperature (K)	292
*a*, *b*, *c* (Å)	23.1656 (12), 7.7041 (3), 16.5664 (8)
β (°)	110.896 (6)
*V* (Å^3^)	2762.1 (2)
*Z*	4
Radiation type	Mo *K*α
μ (mm^−1^)	11.74
Crystal size (mm)	0.2 × 0.2 × 0.1

Data collection	
Diffractometer	XtaLAB AFC12 (RINC): Kappa single
Absorption correction	Multi-scan (*CrysAlis PRO*; Rigaku OD, 2021 ▸)
*T*_min_, *T*_max_	0.479, 1.000
No. of measured, independent and observed [*I* > 2σ(*I*)] reflections	40718, 5446, 4912
*R*_int_	0.056
(sin θ/λ)_max_ (Å^−1^)	0.617

Refinement	
*R*[*F* ^2^ > 2σ(*F* ^2^)], *wR*(*F* ^2^), *S*	0.029, 0.076, 1.02
No. of reflections	5446
No. of parameters	344
H-atom treatment	H-atom parameters constrained
Δρ_max_, Δρ_min_ (e Å^−3^)	0.95, −0.97

Computer programs: *CrysAlis PRO* (Rigaku OD, 2021 ▸), *OLEX2.solve* (Bourhis *et al.*, 2015 ▸), *SHELXL2018/3* (Sheldrick, 2015 ▸), and *OLEX2* (Dolomanov *et al.*, 2009 ▸).

## supplementary crystallographic information

### Crystal data

**Table:**

[Cu_4_Br_6_(C_9_H_11_N_2_O)_2_]	*F*(000) = 2004
*M**_r_* = 1060	*D*_x_ = 2.551 Mg m^−^^3^
Monoclinic, *P*2_1_/*c*	Mo *K*α radiation, λ = 0.71073 Å
*a* = 23.1656 (12) Å	Cell parameters from 8413 reflections
*b* = 7.7041 (3) Å	θ = 2.6–28.6°
*c* = 16.5664 (8) Å	µ = 11.74 mm^−^^1^
β = 110.896 (6)°	*T* = 292 K
*V* = 2762.1 (2) Å^3^	Block, metallic greenish green
*Z* = 4	0.2 × 0.2 × 0.1 mm

### Data collection

**Table:**

XtaLAB AFC12 (RINC): Kappa single diffractometer	4912 reflections with *I* > 2σ(*I*)
Detector resolution: 5.8140 pixels mm^-1^	*R*_int_ = 0.056
ω scans	θ_max_ = 26.0°, θ_min_ = 2.5°
Absorption correction: multi-scan (CrysAlisPro; Rigaku OD, 2021)	*h* = −28→28
*T*_min_ = 0.479, *T*_max_ = 1.000	*k* = −9→9
40718 measured reflections	*l* = −20→20
5446 independent reflections	

### Refinement

**Table:**

Refinement on *F*^2^	Primary atom site location: iterative
Least-squares matrix: full	Hydrogen site location: inferred from neighbouring sites
*R*[*F*^2^ > 2σ(*F*^2^)] = 0.029	H-atom parameters constrained
*wR*(*F*^2^) = 0.076	*w* = 1/[σ^2^(*F*_o_^2^) + (0.0406*P*)^2^ + 3.9895*P*] where *P* = (*F*_o_^2^ + 2*F*_c_^2^)/3
*S* = 1.02	(Δ/σ)_max_ = 0.001
5446 reflections	Δρ_max_ = 0.95 e Å^−^^3^
344 parameters	Δρ_min_ = −0.97 e Å^−^^3^
0 restraints	

### Special details

**Table:**

Geometry. All esds (except the esd in the dihedral angle between two l.s. planes) are estimated using the full covariance matrix. The cell esds are taken into account individually in the estimation of esds in distances, angles and torsion angles; correlations between esds in cell parameters are only used when they are defined by crystal symmetry. An approximate (isotropic) treatment of cell esds is used for estimating esds involving l.s. planes.
Refinement. Refined as a 2-component twin.

### Fractional atomic coordinates and isotropic or equivalent isotropic displacement parameters (Å^2^)

**Table:**

	*x*	*y*	*z*	*U*_iso_*/*U*_eq_	Occ. (<1)
Br2	0.87911 (3)	−0.06317 (11)	0.75247 (5)	0.03317 (18)	0.942 (2)
Br1	0.88670 (3)	0.43944 (11)	0.74718 (4)	0.03271 (18)	0.942 (2)
Br4	0.75698 (3)	0.22558 (9)	0.56292 (4)	0.03737 (17)	
Br3	0.74379 (3)	0.24099 (10)	0.80563 (5)	0.03751 (17)	
Cu1	0.88539 (3)	0.65639 (9)	0.84828 (4)	0.02455 (19)	0.942 (2)
Br6	0.61671 (5)	1.04176 (13)	0.36210 (6)	0.0314 (2)	0.927 (3)
Br5	0.62288 (6)	0.54630 (11)	0.37724 (8)	0.0332 (3)	0.927 (3)
Cu3	0.81423 (4)	0.18899 (12)	0.73163 (7)	0.0435 (3)	0.942 (2)
Cu2	0.61485 (3)	0.82568 (9)	0.46447 (5)	0.0242 (3)	0.927 (3)
Cu4	0.68932 (4)	0.29613 (14)	0.41925 (6)	0.0428 (2)	0.94
O1	0.79475 (19)	0.6363 (6)	0.8272 (3)	0.0420 (10)	
H1	0.779586	0.539265	0.824191	0.063*	
N4	0.6105 (3)	0.7513 (7)	0.5758 (3)	0.0334 (12)	
C5	0.9925 (4)	0.7463 (9)	0.9904 (5)	0.0432 (8)	
N2	0.8871 (3)	0.7459 (7)	0.9601 (3)	0.0313 (11)	
N1	0.9763 (2)	0.6809 (6)	0.9083 (3)	0.0265 (10)	
O2	0.7056 (2)	0.8353 (6)	0.5357 (3)	0.0429 (11)	
H2	0.723439	0.928382	0.550139	0.064*	
N3	0.5234 (2)	0.8094 (6)	0.4310 (3)	0.0276 (10)	
C14	0.5054 (3)	0.7543 (6)	0.4956 (4)	0.0186 (10)	
C15	0.5566 (3)	0.7292 (7)	0.5798 (4)	0.0297 (13)	
C4	1.0527 (3)	0.7781 (9)	1.0393 (5)	0.0428 (9)	
H4	1.063117	0.821780	1.095059	0.051*	
C3	1.0989 (3)	0.7447 (9)	1.0053 (5)	0.0433 (18)	
H3	1.140193	0.765954	1.038035	0.052*	
C13	0.4433 (3)	0.7302 (9)	0.4831 (4)	0.0357 (14)	
H13	0.431362	0.697402	0.528913	0.043*	
C12	0.3997 (3)	0.7558 (9)	0.4020 (5)	0.0370 (16)	
H12	0.358158	0.735100	0.391713	0.044*	
C1	1.0208 (3)	0.6488 (8)	0.8764 (4)	0.0343 (13)	
H1A	1.009791	0.603748	0.820813	0.041*	
C6	0.9388 (3)	0.7732 (8)	1.0175 (4)	0.0327 (14)	
C17	0.6690 (3)	0.7424 (10)	0.6488 (5)	0.0432 (8)	
H17A	0.669363	0.643175	0.685041	0.052*	
H17B	0.675167	0.846878	0.683591	0.052*	
C8	0.8269 (3)	0.7520 (10)	0.9717 (5)	0.0428 (9)	
H8A	0.823678	0.655889	1.007648	0.051*	
H8B	0.823301	0.859334	1.000096	0.051*	
C11	0.4180 (3)	0.8116 (8)	0.3370 (4)	0.0382 (14)	
H11	0.388755	0.832008	0.282335	0.046*	
C9	0.7776 (4)	0.7413 (12)	0.8876 (7)	0.065 (2)	
H9	0.739468	0.795640	0.874160	0.078*	
C2	1.0825 (3)	0.6800 (9)	0.9228 (5)	0.0428 (9)	
H2A	1.112396	0.657735	0.898744	0.051*	
C18	0.7190 (3)	0.7249 (10)	0.6106 (5)	0.0432 (8)	
H18A	0.758582	0.757213	0.653413	0.052*	
H18B	0.721657	0.605151	0.594195	0.052*	
C7	0.9505 (3)	0.8288 (9)	1.1081 (4)	0.0432 (8)	
H7A	0.994219	0.840791	1.138423	0.065*	
H7B	0.934353	0.743263	1.136518	0.065*	
H7C	0.930618	0.938153	1.107883	0.065*	
C10	0.4802 (3)	0.8381 (8)	0.3522 (4)	0.0339 (13)	
H10	0.492326	0.876321	0.307443	0.041*	
C16	0.5438 (4)	0.6878 (10)	0.6593 (4)	0.0495 (18)	
H16A	0.582073	0.671873	0.706658	0.074*	
H16B	0.519781	0.583169	0.650470	0.074*	
H16C	0.521134	0.781528	0.672180	0.074*	
Cu1A	0.8866 (8)	0.837 (2)	0.8496 (12)	0.052 (5)	0.058 (2)
Br1A	0.8769 (7)	0.549 (3)	0.7560 (10)	0.059 (4)	0.058 (2)
Cu3A	0.8077 (7)	0.305 (2)	0.7280 (11)	0.0435 (3)	0.058 (2)
Br2A	0.8834 (8)	0.043 (3)	0.7447 (9)	0.059 (5)	0.058 (2)
Br6A	0.6204 (8)	0.962 (3)	0.3729 (11)	0.056 (4)	0.073 (3)
Cu2A	0.6147 (8)	0.6632 (17)	0.4634 (10)	0.062 (5)	0.073 (3)
Br5A	0.6162 (9)	0.474 (3)	0.3627 (10)	0.063 (5)	0.073 (3)
Cu4A	0.6888 (7)	0.190 (2)	0.4192 (10)	0.0428 (2)	0.06

### Atomic displacement parameters (Å^2^)

**Table:**

	*U*^11^	*U*^22^	*U*^33^	*U*^12^	*U*^13^	*U*^23^
Br2	0.0374 (4)	0.0225 (3)	0.0403 (4)	0.0038 (3)	0.0146 (3)	0.0071 (3)
Br1	0.0417 (4)	0.0279 (4)	0.0331 (4)	−0.0146 (3)	0.0190 (3)	−0.0105 (3)
Br4	0.0293 (3)	0.0509 (4)	0.0253 (3)	−0.0027 (2)	0.0017 (3)	−0.0008 (2)
Br3	0.0309 (3)	0.0501 (4)	0.0380 (4)	−0.0067 (3)	0.0201 (3)	−0.0033 (3)
Cu1	0.0204 (4)	0.0282 (4)	0.0253 (4)	−0.0026 (3)	0.0084 (3)	−0.0037 (3)
Br6	0.0388 (4)	0.0259 (4)	0.0267 (4)	−0.0118 (4)	0.0084 (3)	0.0015 (4)
Br5	0.0387 (5)	0.0225 (4)	0.0389 (5)	0.0045 (3)	0.0143 (4)	−0.0040 (3)
Cu3	0.0419 (5)	0.0373 (5)	0.0589 (6)	0.0010 (4)	0.0271 (5)	−0.0042 (4)
Cu2	0.0202 (4)	0.0300 (4)	0.0218 (4)	−0.0027 (3)	0.0068 (3)	0.0023 (3)
Cu4	0.0415 (5)	0.0386 (5)	0.0389 (5)	0.0043 (4)	0.0028 (4)	0.0039 (4)
O1	0.031 (2)	0.040 (2)	0.056 (3)	−0.0086 (19)	0.018 (2)	−0.003 (2)
N4	0.032 (3)	0.040 (3)	0.024 (3)	0.001 (2)	0.004 (3)	0.001 (2)
C5	0.044 (2)	0.049 (2)	0.0295 (18)	0.0005 (15)	0.0043 (15)	0.0014 (15)
N2	0.031 (3)	0.039 (3)	0.029 (3)	0.002 (2)	0.018 (3)	−0.007 (2)
N1	0.025 (2)	0.029 (2)	0.026 (2)	−0.0008 (18)	0.009 (2)	−0.004 (2)
O2	0.032 (2)	0.049 (3)	0.044 (3)	−0.0079 (19)	0.008 (2)	0.003 (2)
N3	0.028 (2)	0.033 (2)	0.023 (2)	−0.0015 (19)	0.011 (2)	0.0003 (19)
C14	0.023 (3)	0.023 (2)	0.012 (2)	0.002 (2)	0.0084 (19)	0.0002 (19)
C15	0.033 (3)	0.029 (3)	0.030 (3)	−0.002 (2)	0.015 (3)	0.000 (2)
C4	0.033 (2)	0.052 (2)	0.046 (2)	−0.0002 (17)	0.0177 (18)	−0.0032 (18)
C3	0.029 (4)	0.047 (4)	0.045 (5)	−0.003 (3)	0.003 (3)	0.003 (3)
C13	0.036 (3)	0.045 (3)	0.042 (3)	−0.006 (3)	0.034 (3)	−0.001 (3)
C12	0.023 (3)	0.047 (4)	0.039 (4)	−0.002 (3)	0.009 (3)	0.002 (3)
C1	0.028 (3)	0.050 (4)	0.029 (3)	−0.003 (3)	0.015 (2)	−0.006 (3)
C6	0.049 (4)	0.032 (3)	0.026 (3)	0.000 (3)	0.024 (3)	−0.002 (2)
C17	0.044 (2)	0.049 (2)	0.0295 (18)	0.0005 (15)	0.0043 (15)	0.0014 (15)
C8	0.033 (2)	0.052 (2)	0.046 (2)	−0.0002 (17)	0.0177 (18)	−0.0032 (18)
C11	0.027 (3)	0.045 (4)	0.037 (3)	0.004 (3)	0.004 (2)	0.000 (3)
C9	0.031 (4)	0.079 (6)	0.096 (7)	0.001 (4)	0.037 (4)	−0.027 (5)
C2	0.033 (2)	0.052 (2)	0.046 (2)	−0.0002 (17)	0.0177 (18)	−0.0032 (18)
C18	0.044 (2)	0.049 (2)	0.0295 (18)	0.0005 (15)	0.0043 (15)	0.0014 (15)
C7	0.044 (2)	0.049 (2)	0.0295 (18)	0.0005 (15)	0.0043 (15)	0.0014 (15)
C10	0.034 (3)	0.041 (3)	0.030 (3)	0.002 (3)	0.014 (3)	0.005 (3)
C16	0.061 (5)	0.065 (5)	0.023 (3)	−0.006 (4)	0.015 (3)	0.009 (3)
Cu1A	0.058 (11)	0.046 (9)	0.068 (12)	−0.003 (8)	0.039 (10)	−0.004 (8)
Br1A	0.069 (10)	0.064 (12)	0.054 (9)	0.000 (8)	0.034 (8)	−0.002 (7)
Cu3A	0.0419 (5)	0.0373 (5)	0.0589 (6)	0.0010 (4)	0.0271 (5)	−0.0042 (4)
Br2A	0.071 (10)	0.086 (14)	0.036 (8)	0.027 (9)	0.037 (7)	0.009 (8)
Br6A	0.058 (8)	0.065 (11)	0.051 (8)	−0.011 (9)	0.025 (7)	−0.010 (8)
Cu2A	0.081 (12)	0.049 (9)	0.063 (10)	−0.005 (7)	0.036 (9)	−0.006 (6)
Br5A	0.057 (9)	0.104 (15)	0.025 (7)	0.025 (11)	0.011 (6)	0.026 (9)
Cu4A	0.0415 (5)	0.0386 (5)	0.0389 (5)	0.0043 (4)	0.0028 (4)	0.0039 (4)

### Geometric parameters (Å, º)

**Table:**

Br2—Cu1^i^	2.6540 (11)	C14—C15	1.487 (9)
Br2—Cu3	2.4046 (12)	C14—C13	1.390 (8)
Br1—Cu1	2.3739 (10)	C15—C16	1.485 (9)
Br1—Cu3	2.5098 (11)	C4—H4	0.9300
Br4—Cu3	2.6469 (12)	C4—C3	1.400 (10)
Br4—Cu4	2.3990 (12)	C3—H3	0.9300
Br4—Cu3A	2.634 (17)	C3—C2	1.374 (10)
Br4—Cu4A	2.357 (16)	C13—H13	0.9300
Br3—Cu3	2.3987 (11)	C13—C12	1.378 (9)
Br3—Cu4^ii^	2.6288 (12)	C12—H12	0.9300
Br3—Cu3A	2.336 (16)	C12—C11	1.359 (10)
Br3—Cu4A^ii^	2.676 (18)	C1—H1A	0.9300
Cu1—O1	2.008 (4)	C1—C2	1.381 (9)
Cu1—N2	1.964 (5)	C6—C7	1.490 (9)
Cu1—N1	1.992 (5)	C17—H17A	0.9700
Br6—Cu2	2.3878 (11)	C17—H17B	0.9700
Br6—Cu4^iii^	2.5359 (14)	C17—C18	1.512 (11)
Br5—Cu2	2.6357 (11)	C8—H8A	0.9700
Br5—Cu4	2.4092 (14)	C8—H8B	0.9700
Cu2—N4	1.968 (5)	C8—C9	1.455 (12)
Cu2—O2	2.011 (4)	C11—H11	0.9300
Cu2—N3	1.993 (5)	C11—C10	1.387 (8)
O1—H1	0.8200	C9—H9	0.9300
O1—C9	1.448 (10)	C2—H2A	0.9300
N4—C15	1.284 (8)	C18—H18A	0.9700
N4—C17	1.462 (9)	C18—H18B	0.9700
N4—Cu2A	2.016 (15)	C7—H7A	0.9600
C5—N1	1.372 (9)	C7—H7B	0.9600
C5—C4	1.364 (10)	C7—H7C	0.9600
C5—C6	1.479 (11)	C10—H10	0.9300
N2—C6	1.255 (8)	C16—H16A	0.9600
N2—C8	1.473 (8)	C16—H16B	0.9600
N2—Cu1A	1.957 (18)	C16—H16C	0.9600
N1—C1	1.338 (7)	Cu1A—Br1A	2.67 (3)
N1—Cu1A	2.296 (18)	Cu1A—Br2A^iii^	2.34 (3)
O2—H2	0.8200	Br1A—Cu3A	2.40 (2)
O2—C18	1.443 (8)	Cu3A—Br2A	2.62 (2)
O2—Cu2A	2.413 (16)	Br6A—Cu2A	2.77 (2)
N3—C14	1.348 (7)	Br6A—Cu4A^iii^	2.31 (2)
N3—C10	1.351 (7)	Cu2A—Br5A	2.23 (3)
N3—Cu2A	2.284 (17)	Br5A—Cu4A	2.71 (3)
			
Cu3—Br2—Cu1^i^	129.92 (4)	C12—C13—C14	119.0 (5)
Cu1—Br1—Cu3	115.93 (4)	C12—C13—H13	120.5
Cu4—Br4—Cu3	167.35 (5)	C13—C12—H12	120.3
Cu4A—Br4—Cu3A	164.9 (6)	C11—C12—C13	119.5 (6)
Cu3—Br3—Cu4^ii^	159.31 (4)	C11—C12—H12	120.3
Cu3—Br3—Cu4A^ii^	167.0 (3)	N1—C1—H1A	118.8
Cu4^ii^—Br3—Cu4A^ii^	17.7 (4)	N1—C1—C2	122.5 (6)
Cu3A—Br3—Cu4A^ii^	154.2 (5)	C2—C1—H1A	118.8
Br1—Cu1—Br2^iii^	99.38 (3)	C5—C6—C7	118.4 (6)
O1—Cu1—Br2^iii^	97.34 (14)	N2—C6—C5	115.2 (6)
O1—Cu1—Br1	95.65 (14)	N2—C6—C7	126.4 (6)
N2—Cu1—Br2^iii^	104.87 (16)	N4—C17—H17A	110.5
N2—Cu1—Br1	155.74 (16)	N4—C17—H17B	110.5
N2—Cu1—O1	81.9 (2)	N4—C17—C18	106.3 (6)
N2—Cu1—N1	80.5 (2)	H17A—C17—H17B	108.7
N1—Cu1—Br2^iii^	92.51 (13)	C18—C17—H17A	110.5
N1—Cu1—Br1	98.12 (13)	C18—C17—H17B	110.5
N1—Cu1—O1	161.5 (2)	N2—C8—H8A	109.8
Cu2—Br6—Cu4^iii^	116.82 (5)	N2—C8—H8B	109.8
Cu4—Br5—Cu2	131.12 (6)	H8A—C8—H8B	108.3
Br2—Cu3—Br1	104.21 (4)	C9—C8—N2	109.3 (6)
Br2—Cu3—Br4	106.85 (4)	C9—C8—H8A	109.8
Br1—Cu3—Br4	95.25 (4)	C9—C8—H8B	109.8
Br3—Cu3—Br2	124.47 (5)	C12—C11—H11	120.0
Br3—Cu3—Br1	111.79 (4)	C12—C11—C10	119.9 (6)
Br3—Cu3—Br4	110.23 (4)	C10—C11—H11	120.0
Br6—Cu2—Br5	99.05 (4)	O1—C9—C8	112.2 (6)
N4—Cu2—Br6	152.70 (16)	O1—C9—H9	123.9
N4—Cu2—Br5	108.25 (16)	C8—C9—H9	123.9
N4—Cu2—O2	81.6 (2)	C3—C2—C1	118.8 (6)
N4—Cu2—N3	80.6 (2)	C3—C2—H2A	120.6
O2—Cu2—Br6	96.41 (13)	C1—C2—H2A	120.6
O2—Cu2—Br5	95.09 (14)	O2—C18—C17	110.1 (6)
N3—Cu2—Br6	97.65 (14)	O2—C18—H18A	109.6
N3—Cu2—Br5	94.12 (14)	O2—C18—H18B	109.6
N3—Cu2—O2	161.8 (2)	C17—C18—H18A	109.6
Br4—Cu4—Br3^iv^	112.04 (5)	C17—C18—H18B	109.6
Br4—Cu4—Br6^i^	107.88 (5)	H18A—C18—H18B	108.1
Br4—Cu4—Br5	126.29 (6)	C6—C7—H7A	109.5
Br6^i^—Cu4—Br3^iv^	94.15 (4)	C6—C7—H7B	109.5
Br5—Cu4—Br3^iv^	107.49 (5)	C6—C7—H7C	109.5
Br5—Cu4—Br6^i^	103.88 (5)	H7A—C7—H7B	109.5
Cu1—O1—H1	118.7	H7A—C7—H7C	109.5
C9—O1—Cu1	111.4 (4)	H7B—C7—H7C	109.5
C9—O1—H1	109.5	N3—C10—C11	121.1 (6)
C15—N4—Cu2	117.6 (5)	N3—C10—H10	119.5
C15—N4—C17	125.6 (6)	C11—C10—H10	119.5
C15—N4—Cu2A	111.8 (7)	C15—C16—H16A	109.5
C17—N4—Cu2	116.5 (5)	C15—C16—H16B	109.5
C17—N4—Cu2A	114.4 (7)	C15—C16—H16C	109.5
N1—C5—C6	113.1 (6)	H16A—C16—H16B	109.5
C4—C5—N1	121.0 (7)	H16A—C16—H16C	109.5
C4—C5—C6	125.9 (7)	H16B—C16—H16C	109.5
C6—N2—Cu1	117.8 (4)	N2—Cu1A—N1	73.4 (6)
C6—N2—C8	126.1 (5)	N2—Cu1A—Br1A	102.5 (8)
C6—N2—Cu1A	109.5 (7)	N2—Cu1A—Br2A^iii^	158.1 (10)
C8—N2—Cu1	115.6 (4)	N1—Cu1A—Br1A	71.7 (6)
C8—N2—Cu1A	114.9 (7)	N1—Cu1A—Br2A^iii^	117.1 (8)
C5—N1—Cu1	113.3 (4)	Br2A^iii^—Cu1A—Br1A	99.2 (8)
C5—N1—Cu1A	96.5 (6)	Cu3A—Br1A—Cu1A	132.4 (8)
C1—N1—Cu1	127.7 (4)	Br3—Cu3A—Br4	112.7 (6)
C1—N1—C5	118.9 (5)	Br3—Cu3A—Br1A	124.7 (8)
C1—N1—Cu1A	129.7 (6)	Br3—Cu3A—Br2A	108.1 (7)
Cu2—O2—H2	121.1	Br1A—Cu3A—Br4	113.7 (8)
C18—O2—Cu2	110.1 (4)	Br1A—Cu3A—Br2A	101.9 (8)
C18—O2—H2	109.5	Br2A—Cu3A—Br4	88.0 (6)
C18—O2—Cu2A	89.2 (5)	Cu1A^i^—Br2A—Cu3A	116.4 (7)
C14—N3—Cu2	113.5 (4)	Cu4A^iii^—Br6A—Cu2A	127.7 (9)
C14—N3—C10	119.0 (5)	N4—Cu2A—O2	71.2 (5)
C14—N3—Cu2A	100.5 (5)	N4—Cu2A—N3	72.9 (5)
C10—N3—Cu2	127.4 (4)	N4—Cu2A—Br6A	104.3 (6)
C10—N3—Cu2A	127.8 (5)	N4—Cu2A—Br5A	158.7 (9)
N3—C14—C15	114.5 (5)	O2—Cu2A—Br6A	67.2 (5)
N3—C14—C13	121.4 (5)	N3—Cu2A—O2	114.6 (6)
C13—C14—C15	124.0 (5)	N3—Cu2A—Br6A	71.5 (5)
N4—C15—C14	113.6 (6)	Br5A—Cu2A—O2	119.2 (9)
N4—C15—C16	125.4 (6)	Br5A—Cu2A—N3	114.0 (8)
C16—C15—C14	121.0 (6)	Br5A—Cu2A—Br6A	97.0 (8)
C5—C4—H4	120.2	Cu2A—Br5A—Cu4A	116.3 (8)
C5—C4—C3	119.7 (7)	Br4—Cu4A—Br3^iv^	111.8 (6)
C3—C4—H4	120.2	Br4—Cu4A—Br5A	111.4 (8)
C4—C3—H3	120.4	Br3^iv^—Cu4A—Br5A	89.4 (6)
C2—C3—C4	119.1 (7)	Br6A^i^—Cu4A—Br4	123.9 (9)
C2—C3—H3	120.4	Br6A^i^—Cu4A—Br5A	103.4 (8)
C14—C13—H13	120.5		
			
Cu1—O1—C9—C8	−32.9 (9)	C4—C5—C6—N2	175.6 (7)
Cu1—N2—C6—C5	4.5 (8)	C4—C5—C6—C7	−4.4 (11)
Cu1—N2—C6—C7	−175.5 (5)	C4—C3—C2—C1	−0.5 (11)
Cu1—N2—C8—C9	−17.9 (8)	C13—C14—C15—N4	177.2 (5)
Cu1—N1—C1—C2	176.2 (5)	C13—C14—C15—C16	−4.4 (9)
Cu2—N4—C15—C14	3.6 (7)	C13—C12—C11—C10	1.6 (10)
Cu2—N4—C15—C16	−174.7 (5)	C12—C11—C10—N3	0.0 (10)
Cu2—N4—C17—C18	−22.2 (7)	C6—C5—N1—Cu1	3.6 (7)
Cu2—O2—C18—C17	−41.0 (6)	C6—C5—N1—C1	−179.3 (5)
Cu2—N3—C14—C15	4.2 (6)	C6—C5—N1—Cu1A	38.0 (7)
Cu2—N3—C14—C13	−178.1 (4)	C6—C5—C4—C3	179.4 (7)
Cu2—N3—C10—C11	176.0 (4)	C6—N2—C8—C9	170.8 (7)
N4—C17—C18—O2	40.2 (7)	C17—N4—C15—C14	177.2 (6)
C5—N1—C1—C2	−0.4 (9)	C17—N4—C15—C16	−1.0 (10)
C5—C4—C3—C2	0.0 (11)	C8—N2—C6—C5	175.7 (6)
N2—C8—C9—O1	32.5 (10)	C8—N2—C6—C7	−4.3 (11)
N1—C5—C4—C3	0.4 (11)	C10—N3—C14—C15	−179.1 (5)
N1—C5—C6—N2	−5.4 (9)	C10—N3—C14—C13	−1.4 (8)
N1—C5—C6—C7	174.6 (6)	Cu1A—N2—C6—C5	−40.2 (8)
N1—C1—C2—C3	0.8 (10)	Cu1A—N2—C6—C7	139.8 (8)
N3—C14—C15—N4	−5.1 (7)	Cu1A—N2—C8—C9	28.3 (9)
N3—C14—C15—C16	173.2 (6)	Cu1A—N1—C1—C2	128.3 (8)
N3—C14—C13—C12	3.0 (9)	Cu2A—N4—C15—C14	−36.4 (7)
C14—N3—C10—C11	−0.1 (9)	Cu2A—N4—C15—C16	145.4 (7)
C14—C13—C12—C11	−3.1 (10)	Cu2A—N4—C17—C18	18.5 (8)
C15—N4—C17—C18	164.1 (6)	Cu2A—O2—C18—C17	−64.7 (6)
C15—C14—C13—C12	−179.5 (6)	Cu2A—N3—C14—C15	36.2 (6)
C4—C5—N1—Cu1	−177.3 (5)	Cu2A—N3—C14—C13	−146.1 (6)
C4—C5—N1—C1	−0.2 (10)	Cu2A—N3—C10—C11	133.8 (7)
C4—C5—N1—Cu1A	−142.9 (7)		

Symmetry codes: (i) *x*, *y*−1, *z*; (ii) *x*, −*y*+1/2, *z*+1/2; (iii) *x*, *y*+1, *z*; (iv) *x*, −*y*+1/2, *z*−1/2.

### Hydrogen-bond geometry (Å, º)

**Table:**

*D*—H···*A*	*D*—H	H···*A*	*D*···*A*	*D*—H···*A*
O1—H1···Br3	0.82	2.43	3.240 (4)	172
O2—H2···Br4^iii^	0.82	2.40	3.206 (4)	167
C4—H4···Br2^v^	0.93	3.04	3.915 (8)	158
C13—H13···Br5^vi^	0.93	2.99	3.843 (6)	154
C1—H1*A*···Br1	0.93	2.96	3.477 (6)	117
C11—H11···Br3^vi^	0.93	2.95	3.670 (6)	136
C11—H11···Br5^vii^	0.93	3.05	3.790 (7)	138
C9—H9···Br5^viii^	0.93	2.98	3.888 (8)	166
C2—H2*A*···Br2^ix^	0.93	3.09	3.818 (7)	136
C2—H2*A*···Br4^ix^	0.93	2.91	3.658 (7)	139
C18—H18*A*···Br2^iii^	0.97	3.02	3.965 (7)	164
C18—H18*B*···Br4	0.97	3.13	4.086 (7)	169
C7—H7*C*···Br1^viii^	0.96	2.99	3.621 (7)	125
C10—H10···Br6	0.93	2.98	3.482 (6)	115
C16—H16*A*···Br6^viii^	0.96	2.92	3.636 (7)	133

Symmetry codes: (iii) *x*, *y*+1, *z*; (v) −*x*+2, −*y*+1, −*z*+2; (vi) −*x*+1, −*y*+1, −*z*+1; (vii) −*x*+1, *y*+1/2, −*z*+1/2; (viii) *x*, −*y*+3/2, *z*+1/2; (ix) −*x*+2, *y*+1/2, −*z*+3/2.
